# Genome-wide comparative analyses reveal selection signatures underlying adaptation and production in Tibetan and Poll Dorset sheep

**DOI:** 10.1038/s41598-021-81932-y

**Published:** 2021-01-28

**Authors:** Yingyue Zhang, Xianglan Xue, Yue Liu, Adam Abied, Yangyang Ding, Shengguo Zhao, Wenqiang Wang, Liqing Ma, Jijun Guo, Weijun Guan, Yabin Pu, Joram M. Mwacharo, Jianlin Han, Yuehui Ma, Qianjun Zhao

**Affiliations:** 1 Institute of Animal Sciences, Chinese Academy of Agricultural Sciences (CAAS), Beijing, 100193 People’s Republic of China; 2 CAAS-ILRI Joint Laboratory On Livestock and Forage Genetic Resources, Institute of Animal Sciences, Chinese Academy of Agricultural Sciences (CAAS), Beijing, 100193 People’s Republic of China; 3 College of Animal Science and Technology, Gansu Agricultural University, Lanzhou, People’s Republic of China; 4 Qinghai Academy of Animal Science and Veterinary Medicine, Xining, 810016 People’s Republic of China; 5 Animal Husbandry and Veterinary Service of Qinghai, Xining, 810001 People’s Republic of China; 6 International Centre for Agricultural Research in the Dry Areas (ICARDA), P.O. Box 5689, Addis Ababa, Ethiopia

**Keywords:** Biochemistry, Biological techniques, Evolution, Genetics

## Abstract

The identification of genome-wide selection signatures can provide insights on the mechanisms of natural and/or artificial selection and uncover genes related to biological functions and/or phenotypes. Tibetan sheep are an important livestock in Tibet, providing meat and wool for Tibetans who are renown for breeding livestock that adapt well to high altitudes. Using whole-genome sequences with an effective sequencing depth of 5×, we investigated the genomic diversity and structure and, identified selection signatures of White Tibetan, Oula and Poll Dorset sheep. We obtained 30,163,679 Single Nucleotide Polymorphisms (SNPs) and 5,388,372 indels benchmarked against the ovine Oar_v4.0 genome assembly. Next, using *F*_ST_, *ZHp* and XP-EHH approaches, we identified selection signatures spanning a set of candidate genes, including *HIF1A*, *CAPN3*, *PRKAA1*, *RXFP2*, *TRHR* and *HOXA10* that are associated with pathways and GO categories putatively related to hypoxia responses, meat traits and disease resistance. Candidate genes and GO terms associated with coat color were also identified. Finally, quantification of blood physiological parameters, revealed higher levels of mean corpuscular hemoglobin measurement and mean corpuscular hemoglobin concentration in Tibetan sheep compared with Poll Dorset, suggesting a greater oxygen-carrying capacity in the Tibetan sheep and thus better adaptation to high-altitude hypoxia. In conclusion, this study provides a greater understanding of genome diversity and variations associated with adaptive and production traits in sheep.

## Introduction

Sheep (*Ovis aries*) is one of the first domesticated livestock species whose ancestors were primarily distributed in the Fertile Crescent approximately 10,000 years ago^1^. Here, we focus the genome-wide analysis on the Tibetan sheep, an economically important livestock breed in the high altitude Qinghai-Tibetan plateau^2^, which provides meat, milk, wool and skins for nomadic and semi-nomadic people^3^. For instance, the sheep skins can be a source of coat and mattress for local people. In this study, three sheep breeds were used, two local breeds, the Qinghai White Tibetan sheep (BZ) and Oula sheep (OL), and one introduced breed, the Poll Dorset (TST) (Supplementary Table S11). Both OL and BZ are local Tibetan sheep breeds^4,5^. BZ is an ancient breed that is raised for wool and meat production. Notablely, BZ has undergone long-term selection and is characterized by excellent wool quality (long, uniform and elastic fiber) and white coats, which was mainly raised to provide wool for producing the Tibetan-style blanket^6^. TST has been introduced to crossbred with local breeds to improve the meat-yield performance in China. Its distinguishing features include polledness, rapid growth, muscular development and their hardiness and ability to thrive, particularly under hot and dry conditions^7^.

Through long-term natural and artificial selection, livestock leave signals on the genome by which many functional genes can be identified^8^. The identification of selection signals is one of the most important strategies for studying functional genomics^9^. Selection is a vital driving force of evolution. Since the dawn of agriculture, artificial selection has continuously added to, and/or deducted from, existing variations the same way natural selection has impacted biodiversity in nature. Following their domestication and dispersal, the Tibetan sheep gradually adapted to the plateau environment and human requirements. A variety of natural or artificial factors, such as environmental pressure, human migration and socioeconomic practices, have shaped the genome profiles of Tibetan sheep to thrive in the Tibetan environment. The diverse production potentials and extensive adaptations to a wide range of agroecological conditions are believed to result from advantageous mutations and selection pressures, providing the opportunity for identifying selection signatures associated with adaptive and production phenotypes^10,11^.

Next-generation sequencing technologies have been widely used to investigate diverse signatures of selection across a wide range of species^12^. Candidate genes contributing to environmental adaptation have been identified in humans^12–15^, yaks^16^, Tibetan antelopes^17^, gray wolves^18^, dogs^19–21^, cattle^22–25^, pigs^26,27^, chickens^28^, sheep^19,29,30^, goats^31,32^ and Tibetan mastiffs^33^. Similar methodologies have been used to identified genes associated to production traits and the impact of domestication in livestock chickens^34,35^, cattle^36^, goats^32,37^, dogs^38^, rabbits^39^, salmon^40^ and sheep^25,37,41,42^. In sheep, several studies on selective signatures have identified genes linked to domestication and productive traits. Kijas et al.^43^ identified *BMP2*, a gene associated with bone morphology and body shape, in a selection signature analysis of a large number of globally distributed sheep breeds. Lv et al. identified genomic regions and genes associated with environmental adaptation based on climate variables and genomic data of a larger set of native sheep breeds from a worldwide range of geographic areas and climate^10^. Using F_ST_ analysis, Zhang et al. conducted a genome-wide selection signal detection in five sheep breeds and identified genes related to important traits. For instance, *RXFP2*, *GHR* and *ASIP* are associated with the shape of horn, growth, and lipid metabolism^44^. Genome-wide selective signature analysis of resequencing data from 77 Chinese domestic and three wild sheep revealed several novel candidate genes related to extreme environmental adaptation. The study detected the selected regions mostly spanned milk- and meat-related QTLs, reflecting human demands for milk and meat during sheep domestication. The *SOCS2* was identified as an important candidate gene in Tibetan sheep for responding to high-altitude stress through the regulating the *EPO* gene in the HIF-1 pathway^45^.

Blood physiological parameters indicate not only health and metabolic conditions but also shed light on adaptation to specific environments^46,47^ Long-term severe reduction in oxygen availability at high altitudes contributes to physiological adjustments in response to hypoxia adaptation^48,49^. Dramatic hematological differences have also been observed between species living at high-altitudes and the ones at low altitudes^50–52^.

To explore the genetic variance and genetic structure as well as to identify candidate regions and genes related to important traits, we resequenced the whole genomes of 47 sheep from two Tibetan sheep breeds and an imported breed and performed a within and between breed comparative selection signature analysis. In addition, we also calculated indices for several blood parameters to get insights on the physiology of adaptation. The study is aimed to provide a theoretical basis for improving economically important traits in Tibetan local sheep breeds and to further provide insights on the mechanisms underlying the adaptation to high altitudes.

## Results and discussion

### Data production

#### Blood physiological indices and parameters

To obtain deeper insights on the physiology of adaptation, we measured nine blood physiological parameters including WBC, RBC, HGB, HCT, MCV, MCH, MCHC,RDW-SD and LYM for 186 individuals from three breeds. The levels of MCH and MCHC for the two local Tibetan sheep breeds were higher compared to those of Poll Dorset (*P* < 0.01; Supplementary Fig. S1). It may indicate a higher oxygen-carrying capacity in the blood of Tibetan sheep due to the fact that Tibetan sheep are long-term inhabitants of high altitude environments. Though it remains to be ascertained, it has been suggested in other literature that blood physiological parameters such as hemoglobin levels often play a major role in mediating adaptive response to plateau hypoxia. It has been reported that physiological adaptation to higher altitudes in yak has been attributed to increases in RBC and blood oxygen affinity but decreases in MCV^52^. Previous studies revealed higher levels of MCH and MCHC in sheep^29^ and horse^53,54^ from the Tibetan plateau compared with animals in low altitudes. Nevertheless, genome-wide association analysis (GWAS) between genomic loci and the blood physiological parameters was not performed due to the relatively small number of sheep that were resequenced. MCV was found to divergent among humans living at high altitudes and at sea level, with significantly higher values associated with humans living at higher altitudes^55^.

#### SNP identification and annotation

To characterize the genetic variance of three sheep breeds, the whole-genome resequencing of 47 individuals was performed on an Illumina HiSeq 2500 system. More than 860 Gb, 2 × 125 bp paired-end reads were generated and aligned using Burrows-Wheeler Aligner (BWA)^56^ software against the sheep reference genome assembly. Over 99.38% of the sequence reads were mapped to the reference genome. We achieved an average sequencing depth of 5.84× for each breed, and more than 99.33% of the total clean reads mapped against the sheep reference genome, indicating high-quality sequences were obtained in this study (Supplementary Table S1). We ultimately identified 30,163,679 SNPs and 5,388,372 indels for subsequent analysis using SAM tools v0.1.19^57^. All raw data were deposited into a NCBI BioProject section under accession number PRJNA675420.

To detect genomic regions under selection, we used SAMTools v0.1.19^40^ to collect summary information from the input BAM (Binary Alignment Map) files and calculated the probability of the data given for each possible genotype and stored the probabilities in the BCF (Binary Variant Call Format) file. BCF tools was applied to the prior data for SNP calling and to convert the data to VCF (Variant Call Format) files, which can be used in subsequent analysis^58^. We obtained over 30 million high-quality SNPs from the 47 resequenced sheep, most of which were located in intergenic regions (18.83 million, 62.06%), and only 0.71% (214,098) were located in exonic regions (Supplementary Table S2). A total of 62,154 non-synonymous (29.03%) and 81,504 synonymous, SNPs (38.07%) were localized within exons, resulting in a non-synonymous/synonymous ratio of 0.762 (Supplementary Table S3). We identified 5,388,372 indels, of which 44,557 were exonic, and the frequency of indels decreased as their sizes increased (Supplementary Table S4). The proportion of indels in intergenic, intronic, and exonic regions were 60.48% (3,258,720), 35.0% (1,885,711) and 0.83% (44,557), respectively (Supplementary Table S5).

#### Genetic diversity and variation

To gain insights on genetic diversity of the three sheep breeds, the expected heterozygosity (H_E_), observed heterozygosity (H_O_) and minor allele frequency (MAF) were estimated based on genotype frequencies (Supplementary Table S6). The estimates of H_E_, H_O_ and MAF among the three sheep breeds were 0.22, 0.19 and 0.15, respectively. These values are lower than those observed in free ranging and random mating Egyptian Barki sheep populations that are adapted to a hot arid environment^59^. OL displayed a lower level of genetic diversity compared with TST and BZ. TST had the lowest inbreeding coefficient (F_IS_ = 0.14), while OL had the highest value of inbreeding coefficient (F_IS_ = 0.20). This corresponds to the breed characteristics and breeding history of OL. OL is a breed with a long-term artificial selection for meat production trait and has been bred in a considerable isolated environment, which result in the low genetic diversity but higher inbreeding coefficient. In future, a resonable breeding plan for OL breed should be implemented to conserve its genetic diversity.

### Phylogenetic analysis

#### Principal component analysis (PCA)

**Figure 1:**
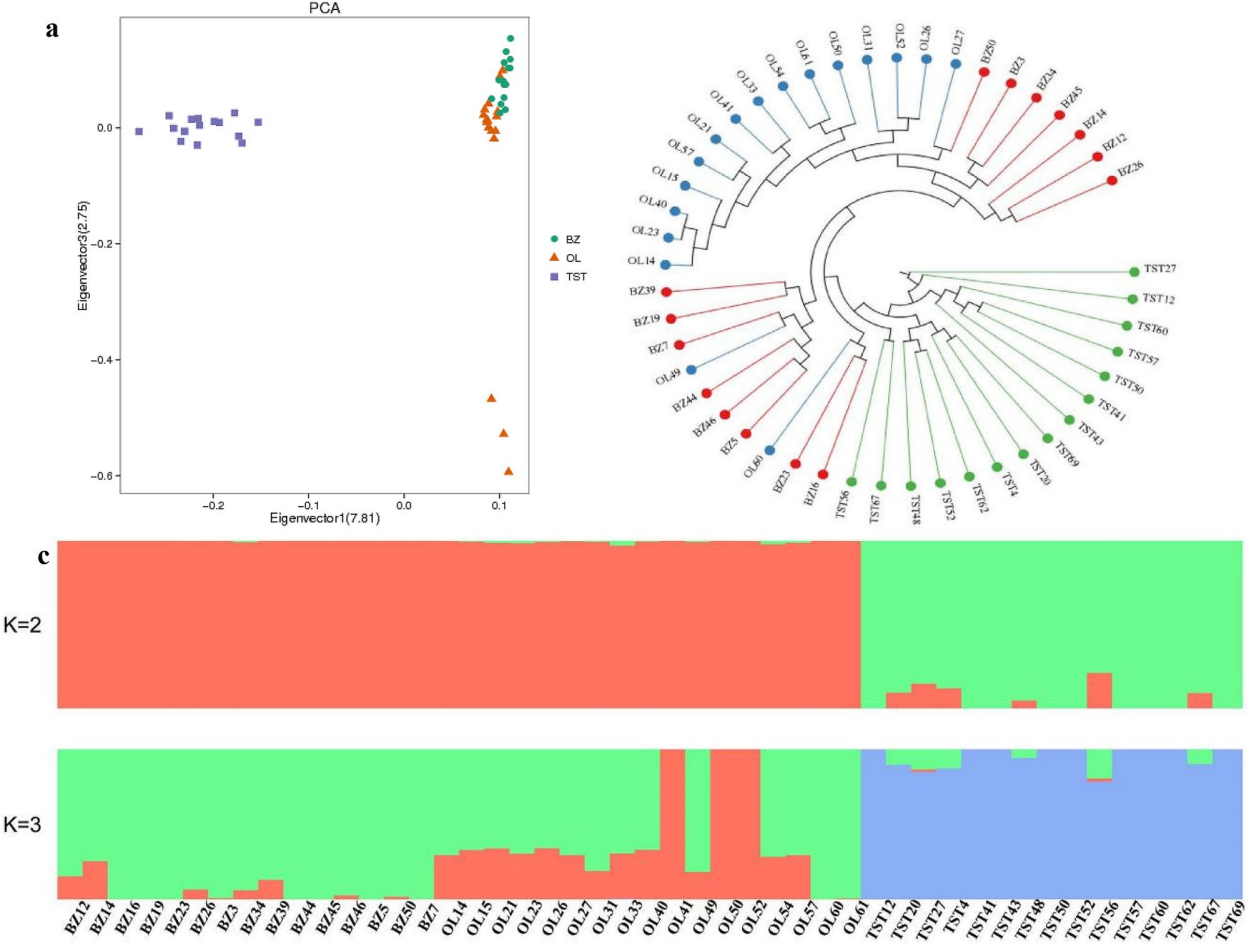
Population genetics analyses of samples. (**a**) Principal component analysis (PCA) results of three sheep breeds. (**b**) NJ tree constructed using p-distances between individuals. (**c**) Population genetic structure of the sheep inferred from the program FRAPPE v1.1. The length of each colored segment represents the proportion of the individual genome inferred from ancestral populations (K = 2 or 3).

To examine genetic relationships among and within the three breeds, we first performed PCA analysis^60^. The first eigenvector clearly distinguished the TST breed from BZ and OL, and the second eigenvector distinguished the two Tibetan sheep breeds (Fig. 1a). In general, and as expected, the PCA distinguished the introduced TST breed from the two local Tibetan breeds (BZ and OL) which showed close genetic proximity. Unexpectedly, 14 individuals of BZ clustered together with OL, suggesting very close genetic relationships due to either close geographic proximity or deliberate intercrossing. Although OL and BZ are classified as Tibetan sheep breeds category, they have distinct morphological characteristics and breeding histories. BZ is an ancient breed bred by Tibetans and is adapted to the high-altitude Qinghai-Tibet plateau environment. The breed is known for outstanding wool quality but low meat production. OL originated from local Tibetan sheep and wild sheep^61^.

#### Phylogenetic tree

To further investigate the genetic relationships of the three sheep breeds, a phylogenetic tree was constructed with the filtered SNP set using the neighbor joining (NJ) algorithm. The NJ tree clustered the three studied breeds into separate genetic groups confirming their genetic distinction (Fig. 1b). Consistent with the PCA results, 14 individuals of OL clustered with the BZ, confirming close genetic relationship or gene flow between the two breeds due to close geographic proximity.

#### Population genetic structure

To determine the proportion of shared genetic ancestry and/or levels of admixture, we used Frappe^62^ to explore population genetic structure, at 2 ≤ *K* ≤ 3 corresponding to the maximum number of breeds analysed in the study and following the results of the PCA and NJ phylogenetic tree. At *K* = 2, the three sheep breeds are genetically divided into the TST breed and the two Tibetan breeds. At *K* = 3, majority of BZ individuals separate from the OL ones, except for three OL individuals that remain clustered with BZ (Fig. 1c). Furthermore, with the exception of only three individuals of OL, the rest had the similar genetic background of BZ. Consistent with the results of PCA and NJ, the admixture analysis further confirms with higher resolution, the intermixed genetic makeup of the two Tibetan sheep breeds. Long-term mating of OL and other Tibetan sheep breeds with non-desirable meat traits is a common practice for improvement of the meat production, which result in gene flow of these breeds.

#### Linkage disequilibrium (LD) analysis, runs of homozygosity (ROH) and pairwise sequentially Markovian coalescent (PSMC)

**Figure 2:**
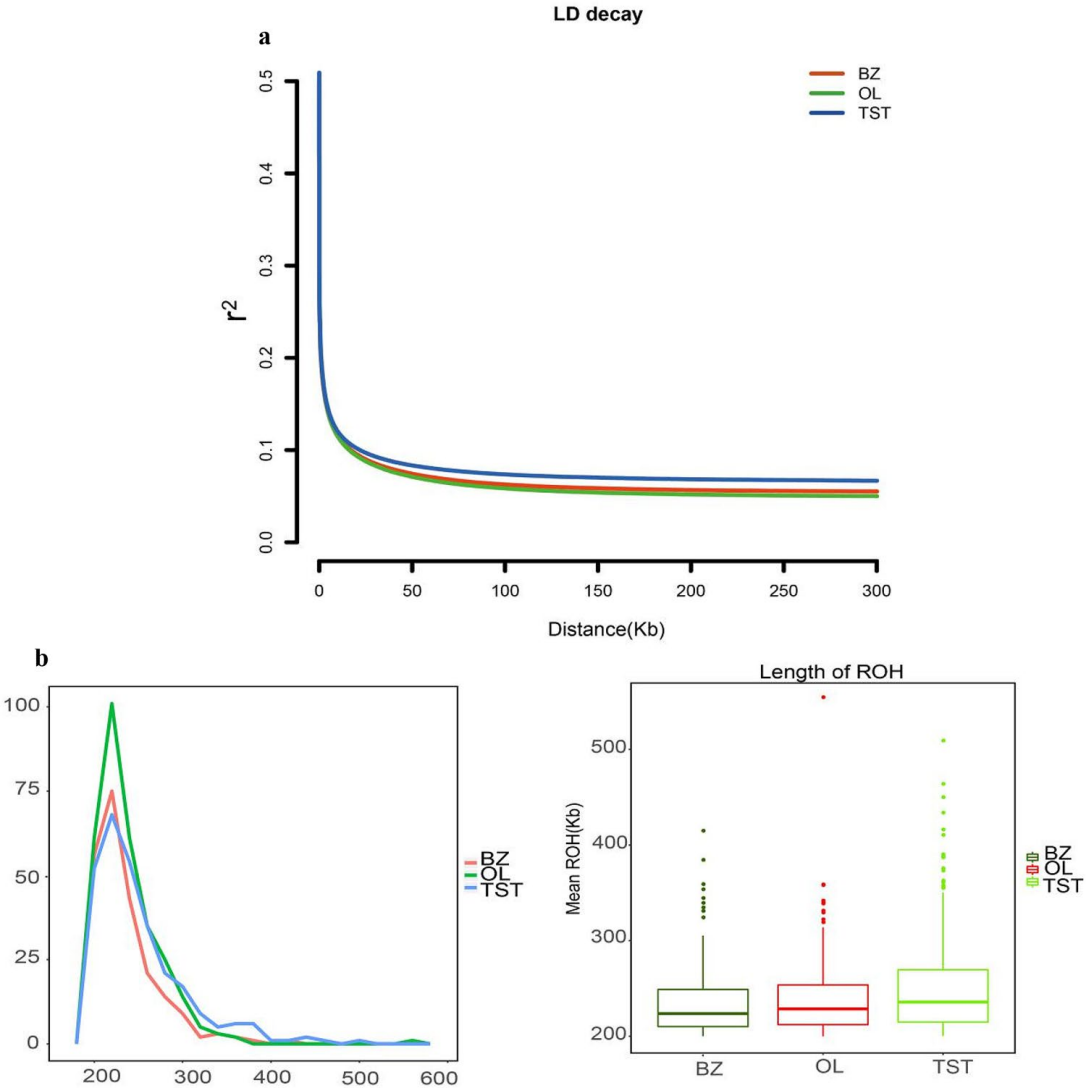
The population genetic and demographic dynamics of each Sheep breed. (**a**) Decay of linkage disequilibrium (LD) in the Chinese native sheep breeds, with one line per breed. (**b**) The length and distribution of ROH provide information regarding.

To better understand the population genetic and demographic dynamics of each breed, we used PopLDdecay software to explore genome-wide patterns of LD in each breed-group invoking the default parameters. The LD patterns and ROH analysis result are presented in Fig. 2. The OL and BZ breeds had lower LD values, suggesting a relatively early origin of the Tibetan sheep breeds. The TST group had higher LD value, indicating that it was probably derived from a relatively small ancestral population or long-term selection has retained relatively large LD blocks in its genome. The length, frequency and distribution of ROHs can provide useful information regarding an animal’s ancestry and the history of the population^63^. Selection process may give rise to high level of homozygosity, also called runs of homozygosity (ROH). The size, frequency and distribution of ROHs in the genomes from three sheep breeds were investigated. A variety of lengths of ROHs existed in the genomes of the three sheep breeds (Fig. 2). Consistent with the LD findings, ROH distribution analysis showed that the average size and frequency of ROHs in TST were relatively higher than those of BZ and OL, respectively.

**Figure 3:**
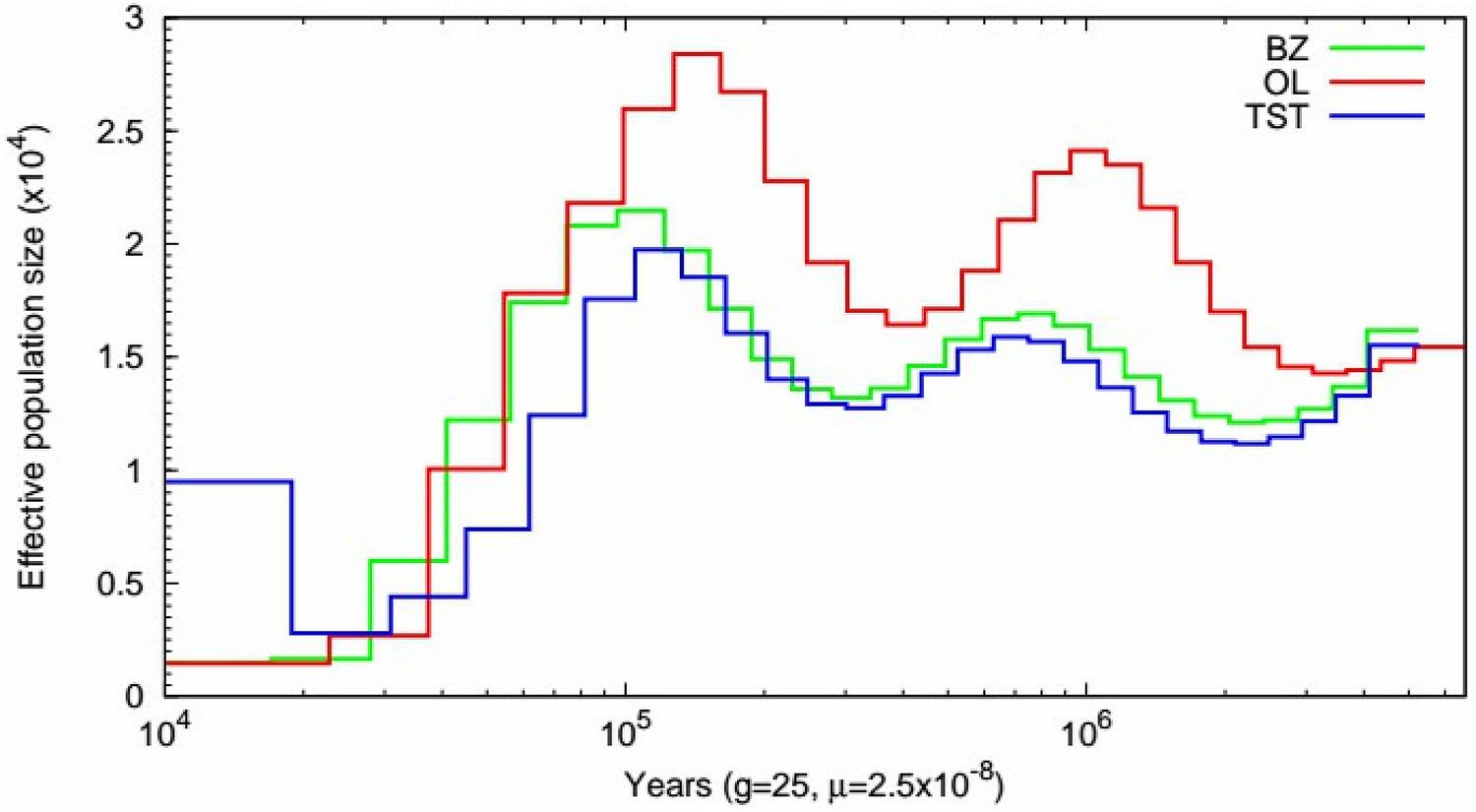
Demographic history of the sheep populations. PSMC analysis results for the representative individuals sequenced at a high read coverage (~ 42×) exhibit inferred variations in Ne over the last 10^6^ years.

The PSMC approach can estimate trajectories in changes in genomic effective population sizes (N_E_) over considerable time periods for the ancestor of specific population^64^. The change in the trends in N_E_ for the three breeds was the same 20,000 years ago (Fig. 3). There were two peaks in the trends in N_E_, one at 300,000 years and the other about one million years ago. At 100,000 years ago, population size shrinkage was observed in the three breeds. OL and BZ showed a strong correlation since 100,000 years ago. At 20,000 years, the effective population size of TST increased^65,66^.

### Genome-wide selection signature analysis

**Figure 4:**
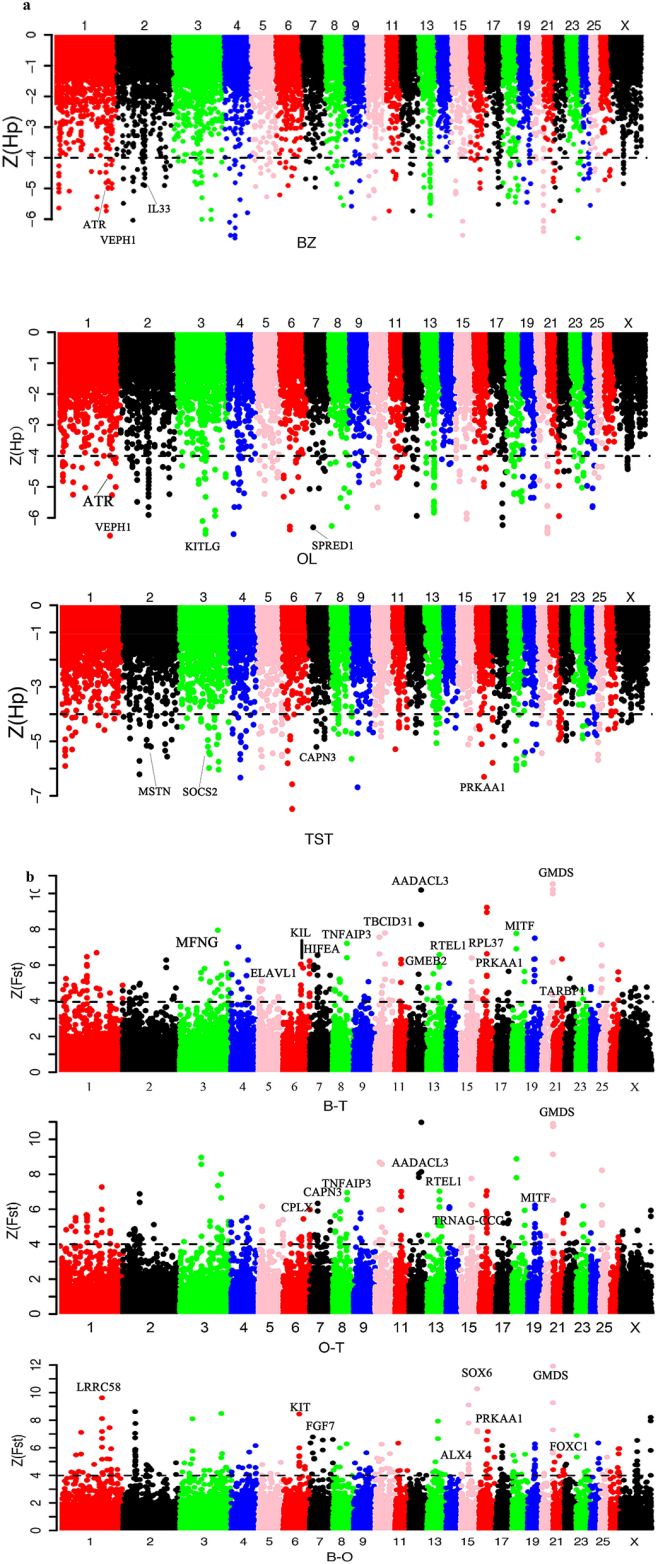
Analysis of the signatures of positive selection in the genome of samples. Genomic landscape of the (**a**) ZHP values and (**b**) ZF_ST_ values.

Over the past a few years, the identification of selection signatures has led to significant improvements in understanding the genetics of important economic and adaptive traits^67^. Selection signatures have been used to identify differentially selected genes and genomic regions in different populations. To better understand the underlying genetics of phenotypic traits, production traits and adaptation among the three sheep breeds, three methods (*F*_ST_, ZHp and XPEHH) were used to identify selection signatures. First, we measured the heterozygosity^68^ (*Hp*) in 100-kb windows with half-step sliding along the genomes of the three breeds. The candidate regions defined by ZHp spanned 279, 377 and 450 genes for BZ, OL and TST, respectively. Of these genes, 45 genes were shared across all the three breeds (Supplementary Table S10). The fixation index^69^ (*F*_*ST*_) and the Cross-population Extended Haplotype Homozygosity (XPEHH)^70,71^ tests involving BZ-OL, OL-TST and BZ-TST, were also performed in 100-kb windows with 50-kb sliding step. Putative selection targets were extracted from the extreme ends of the distributions by applying a ZHp < − 4, Z*F*_*ST*_ > 4 cut-off thresholds and top 1% of SNPs for XPEHH. The distributions of the ZF_ST_, ZHp and XPEHH scores suggested the evidence of putative selection in the genomes of the examined breeds (Fig. 4, Supplementary Fig. S2). The fixation index (F_ST_) reflect the degree of population difference, which has been wildly applied to scan the selective sweeps. Several genomic regions with high F_ST_ values were detected, such as *GMDS*, *KIT* and *PRKAA1*. Up to 421 genes were positively selected for F_ST(OL-TST)_ comparison, 359 genes for F_ST(BZ-TST)_ comparison and 344 genes for the F_ST(BZ-OL)_ comparison. In addition, XP-EHH method, which tracks down long-range haplotypes with high frequency, as are indicatives of chromosomal regions under recent selection, was applied for detecting the selective signatures. A total of 750 genes were spanned by the candidate regions identified by XP-EHH method among the three comparisons performed: 331 genes were positively selected for XPEHH_(OL-TST)_ comparison, 105 genes for XPEHH_(BZ-TST)_ comparison and 314 genes for the XPEHH_(BZ-OL)_ comparison. Compared to other Tibetan sheep breeds, large body size is its strking features for OL. In addition, coat colour pattern and fiber trait of OL and BZ are different. In the comparison of two local Tibetan breeds (BZ and OL), *KIT*, *SOX6*, *PRKAA1* and *FGF7* were identified as putative candidate genes through the genome-wide analysis of selective sweep. Previous studied showed *KIT* and *FGF7* are associated with coat colour traits and wool growth development,respectively^72,73^. *SOX6* is related to muscle development in chicken and zebrafish^74^. *PRKAA1* functions as a cellular energy sensor under ATP-deprived conditions such as those experienced in hypoxia, suggesting a biologically-plausible role for the PRKAA1 (AMPKa1)-mTOR pathway in metabolic responses to hypoxic environments. To improve the confidence in the selected outlier windows of putative genomic regions under selection, we identified 88 overlapped genes, which may contribute to growth and development, behavioral, immune, and morphological differences among breeds using the criterion of Fst > 4 and a top 1% outlier of XP-EHH. The list of non-synonymous mutations of overlapping genes in both Fst and XP-EHH methods was showed (Supplementary Table S7).

We conducted a Gene Ontology analysis and Kyoto Encyclopedia of Genes and Genomes of candidate genes identified in the three breeds by individual selection methods (Supplementary Table S8, Supplementary Table S9). It revealed enrichments in 529 biological processes GO terms, 98 molecular functions GO terms, and 65 cellular components GO terms based on the *P* value of 5%. A few genes were related to reproduction (GO: 0000003), the immune system (GO: 0002682), embryo development (GO: 0009790, GO: 0048598), growth (GO: 0060173), hair development (GO: 0001942), nervous-system development (GO: 0050951, GO: 0007632) biological processes, as well as interleukin (GO: 0070555), skeletal-system development (GO: 0001501), heart development (GO: 0021591), response to hypoxia (GO: 0097411), inflammatory response (GO: 0045088) and sensory perception (GO: 0050951). All the enriched functional terms had a significant enrichment score (*P *value < 0.05). These findings implied genes relating to growth and development, immune response, adaptation for hypoxia and wool traits could be targets of selection in BZ and OL during domestication and breeding improvement.

#### Putative sweeps related to high-altitude adaptation

Tibetan sheep are capable of surviving in high-altitude cold and dry environments ranging from 2900 to 4500 m above sea level. High-altitude adaptation is the most significant feature for Tibetan sheep compared to sheep populations from lowland. We therefore focused on the candidate genomic regions spanning genes related to hypoxia adaptation. Several genes associated with high-altitude adaptation were detected in our selection signature analysis, including *HIF1A*, *ATR*, *SLC24A4*, *PPA2*, and *ROCK2*. Recent studies have indicated that the regulation of *HIF1A* stability and transactivation activity involves several proteins and their well-coordinated interaction, raising the possibility that a wide range of control mechanisms could be involved in mediating physiological responses to oxygen availability^74–77^. *ATR*, which functions in DNA repair, was reported to regulate the expression of hypoxia-inducible factor (HIF)-1 alpha and confers hypoxia tolerance^78^. Here, we found that *ATR* was detected in candidate region on chromosome 1 in BZ and OL but not in TST, suggesting its involvement in high-altitude adaptation in the two Tibetan breeds. *SLC24A4* (sodium/potassium/calcium exchanger 4) is located in the classical HIF-1 pathway, which plays a central role in regulating cellular responses to hypoxia^45^. A previous study suggested that *PPA2* is a cardiomyopathy-associated protein that plays a physiological role in mitochondrial function^78^. Another study suggested that polymorphisms and the haplotype of *ROCK2* are associated with high-altitude essential hypertension in the Ladakhi Indian population that resides at a high-altitude^79^.

#### Candidate genes related to meat and wool traits

Genes associated with meat quality, including *CAPN3*^34,36,38^, *SOX6*^72–76^*, FGF5*^80–83^, *FGF7* and *VEPH1*^84,85^*,* were identified in several candidate genomic regions under selection. Calpain 3 (*Capn3*), a skeletal muscle-specific member of the calpain family, was suggested to be related with muscle growth in cattle^36,71,77,86^ and chicken^34,87^. Fibroblast growth factor (FGF) 5 regulates the development and periodicity of hair follicles, which is related to wool or cashmere growth in cats, sheep and goats^80,81,83^. *FGF5* knockout sheep or goats have significantly increased hair follicles, fiber length and growth rates^80^. As a famous local breed due to its superior wool production performance in the Qinghai-Tibetan Pleatau, BZ has longer fiber compared to OL and TST, which have undergone long-term selection for wool trait. Fiber from BZ is used by local Tibetans to weave blankets. *VEPH1* (ventricular zone expressed PH domain containing 1) is associated with lipid metabolism and has been reported to be involved in diabetes in humans, fat deposition in pigs and rump fat thickness and carcass traits in sheep. These findings suggest that *VEPH1* may function to regulate growth and overall body size in mammals^80,81^.

#### Candidate genes related to disease and immunity

Selective sweep analysis also identified candidate genes associated with disease resistance and immunity, including *GMDS*^88^, *GMEB2*^89^, *TNFAIP3*^90,91^ and *TET2*^92^ in several candidate genomic regions in three sheep breeds. A deficiency in *GMDS* leads to escape from NK cell-mediated tumor surveillance through the modulation of TRAIL signaling^93^. The variant in *TNFAIP3* has been associated with systemic lupus erythematosus, an autoimmune disease. *B*2*M* has been implicated in antigen processing and presentation of peptide antigens via MHC Class Ib^88,89^.

### Candidate genes related to reproduction and body size

The thyroid-stimulating hormone receptor gene (*TSHR*) was identified in our study by high Z*F*_*ST*_ and XPEHH values in one candidate region. *TSHR* has been reported to play crucial roles in metabolic regulation and the photoperiod control of reproduction in vertebrates such as domestic chickens^35,94^ and sheep^79^. Whole genome scans revealed a distinct selective sweep located at the locus for *TSHR* in domestic chickens, which suggested *TSHR* is likely related to seasonal reproduction in vertebrates. *ARFRP1* encodes androgen receptor and is essential for prostate gland development and reproduction^91^. In our study, the *ARFRP1* gene was found in a candidate selection sweep region in the Tibetan breeds. *HOXA10* is a well-known transcriptional factor and is regarded as one of the most promising candidate genes to play major roles in endometrial differentiation and development through establishing the conditions required for implantation and normal pregnancy maintenance^92,95^. It has been widely studied in humans, mice and other species. In our study, *HOXA10* occurred in a candidate region defined by a strong selection signature in the Poll Dorset and we suggest that this gene could be an important factor underlying reproductive performance.

## Conclusion

Our study provides comprehensive insights into the phylogenetic relationship among BZ, OL and TST sheep. Analysis of blood parameters revealed higher levels of mean corpuscular hemoglobin measurement and hemoglobin concentration in the Tibetan sheep, which may contribute to their adaptation to high altitudes. We identified several candidate genes under selection in three sheep breeds, which exert their essential roles in hypoxia adaptation, growth and development, wool trait as well as other traits. The large number of genetic variants identified in this study provides the opportunity to further explore the genetic diversity in sheep and the genetic basis underlying different phenotypes. Our results contribute to the growing knowledge base on genomics of adaptation in livestock and provide valuable information for future studies on genotype–phenotype relationships and the improvement of sheep breeding.

## Methods

### Sample collection

Three sheep breeds were sampled from the northeast part of Qinghai province as follows: White Tibetan sheep (BZ) from Qilian County, Oula sheep (OL) from Henan County and the introduced Poll Dorset sheep (TST) from Haiyan County. The average altitude of these counties is 3500 m above sea level. We collected two sets of 5 ml jugular venous blood samples from each animal. The first set was collected in coagulant tubes while the second set was collected in anticoagulant (4% (w/v) sodium citrate) tubes. The latter were stored at − 80 °C until further processing. We carried out a test on blood physiological parameters and calculated indices for White (WBC) and Red Blood Cell counts (RBC), Hemoglobin concentration (HGB), Hematocrit (HCT), mean corpuscular volume (MCV), Mean corpuscular hemoglobin measurement (MCH) and Mean corpuscular hemoglobin concentration (MCHC) from 186 unrelated individuals (63 OL, 53 BZ and 70 TST). All blood parameter measurements were performed within three hours following sampling with Vet Autoread Blood Analyzer (IDEXX, America) . From the 186 individuals, samples from 47 three-year old individuals (15 BZ, 15 OL and 17 TST) were selected at random and resequenced.

### Ethic statement

All experimental procedures used in this study were approved by the Animal Care and Use Committee of the Institute of Animal Sciences of Chinese Academy of Agricultural Sciences (CAAS) and conducted in accordance with animal welfare and ethics guidelines of the academy.

### DNA isolation and sequencing

DNA was extracted from blood using the QIAamp DNA Blood Mini Kit (Qiagen) according to the manufacturer’s instructions. The extracted DNA was electrophoresed through a 2% agarose gel and stained with ethidium bromide to assess overall quality. The DNA concentration was determined using the Quant-iT PicoGreen dsDNA Reagent Kit (Thermo Fisher Scientific, USA) according to the manufacturer’s instructions. Libraries were prepared using Illumina kits with an insert size of approximately 500 bp. Two × 125-bp paired-end sequencing was carried out on an Illumina HiSeq 2500 instrument (Illumina; CA, USA). To produce “high quality clean data” from the “raw data,” filtration was performed as follows: (1) Removal of adapter reads: An adapter read is defined as one that includes more than 5 adapter base pairs. The paired-end read was discarded if either read contained adapter bases. (2) Removal of low-quality reads: If more than 50% of the bases in a read were low-quality, defined as a base quality of less than or equal to 5, we designated the read as a low-quality read and removed it from the raw FASTQ data. The paired reads were discarded when either read contained more than 50% low-quality nucleotides. (3) Removal of reads with more than 5% unknown bases (Ns): The paired reads were discarded when either end of one read contained more than 5% unknown bases. All bioinformatic analyses were based on the clean reads resulting from Illumina quality control filter.

### Read alignment and variant calling

The Burrows–Wheeler aligner (v0.7.9a; MEM model) was used to map the clean reads to the Oar_4.0 *Ovis aries* genome assembly. Duplicate reads were removed from individual alignments using the Picard MarkDuplicates tool (v1.115). Reads mapping to more than two genomic locations were filtered out. The Genome Analysis Toolkit (GATK) Haplotype Caller protocol was used to call SNPs and indels via local re-assembly of haplotypes. The SNPs and indels were filtered prior to analysis with the GATK Variant Filtration protocol. The filter settings were as follows: QD < 10.0, ReadPos RankSum < − 8.0, FS > 10, QUAL < 30, DP < 4.

### Variant annotation

ANNOVAR was used to assign SNPs and indels based on gene models from GFF annotation.

### Phylogenetic analysis and demographic dynamics

Principal component analysis (PCA) was performed using the EIGENSOFT package on the filtered SNP set. The top four principal components accounting for variation in the dataset were identified. A phylogenetic tree was constructed with the filtered SNP set with the neighbor joining (NJ) algorithm. PHYLIP^60^ was used to generate genetic distance matrix which were used in MEGA^96^ to construct the phylogenetic tree. Frappe (version 1.1) was run using the filtered SNP set to analyze population structure. The filtered SNP set was also used to estimate genome-wide linkage disequilibrium (LD). The LD decay was calculated with the PopLDdecay using default parameters. We performed ROH detection using PLINK v1.09 based on the identified high-quality SNP sites with the following parameters: “--homozyg --noweb --sheep --allow-no-sex --homozyg-window-kb 5000 --homozyg-window-snp 50 --homozyg-window-het 1 --homozyg-window-missing 5 --homozyg-snp 10 --homozyg-kb 200 --homozyg-density 50 --homozyg-gap 1000”.

### Selective sweep analysis

Allele counts and allele frequencies at filtered SNP sites were used to detect genomic regions that may have been affected by selective breeding, migration and adaptation in the genomes of the study sheep breeds. First, the average pooled heterozygosity (HP) was calculated in 100-kb windows with a sliding step of 50 kb for each breed. The resulting distribution of HP scores was then Z-transformed into ZHp values. Second, the fixation index (*F*_*ST*_) between the breeds was calculated using VCFtools (0.1.12b) to evaluate genetic differentiation. We averaged the *F*_*ST*_ across 100-kb windows, with a sliding frame of 50 kb at a time and Z-transformed the resulting distribution. The parameters for the VCFtools program were as follows: “--fst-window-size 100,000 --fst-window-step 50,000”. Putative selection targets were extracted from the extreme tail ends of the distribution by applying a Z (*F*_ST_) value > 4 and the corresponding ZHp < − 4 as cut-off thresholds.

Additionally, cross population extended haplotype homozygosity (XP-EHH) method was used to scan the selection signals. We compared the extended haplotype homozygosity (EHH) among the three sheep breeds (OL vs. TST, BZvs TST and BZ vs. OL) using XP-EHH statistic implemented in the rehh package. Candidate regions with a *P* value ≤ 0.01 were considered as signals of selection in the test.

### Functional enrichment analysis

Enrichment analysis was performed to determine functional clusters for the candidate genes using gene ontology (GO) and KEGG pathway analysis. GO enrichment analyses was performed using OmicShare tools (http://www.omicshare.com/tools). First, all candidate genes were mapped to GO terms in the GO database (http://www.geneontology.org/) and the significant number of genes for every term were determined using a *P* value ≤ 0.05 as the threshold. The GO terms satisfying this criterion were defined as significantly enriched GO terms for the candidate genes. Second, all candidate genes were mapped to GO terms in the KEGG pathway database (http://www.genome.jp/kegg/ko.html). KEGG pathway-enrichment analysis identified significantly enriched metabolic and/or signal transduction, pathways for genes found in the candidate regions compared to the whole genome^97–99^. The calculations and criteria for determining the significantly enriched pathways was the same as that used in GO analysis.

## Supplementary Information

Supplementary Information

## Data Availability

The datasets generated in this paper can be found at Sequence Read Archive: PRJNA675420.
