# Supplementary material for: Genome-wide comparative analyses reveal selection signatures underlying adaptation and production in Tibetan and Poll Dorset sheep
Source: Sci Rep. 2021 Jan 28;11:2466. doi: 10.1038/s41598-021-81932-y (PMC7844035; doi:10.1038/s41598-021-81932-y)

**Genome-wide comparative analysis reveal selection signatures underlying adaptation and** **production in Tibetan and Poll Dorset sheep**

Yingyue Zhang^1,2＃^, Xianglan Xue^1,2＃^, Yue Liu^1,2^, Adam Abied ^1^, Yangyang Ding^1,2^, Shengguo Zhao^3^, Wenqiang Wang^1,3^, Liqing Ma^4^, Jijun Guo^5^, Weijun Guan^1,2^, Yabin Pu^1,2^, Joram M. Mwacharo^6^, Jianlin Han^1,2^, Yuehui Ma^1,2*^, Qianjun Zhao^1,2*^

**Supplementary Table S1.** Whole genome resequencing for 32 local Tibetan sheep and 15 introduced sheep

| **Sample** | **BZ** | **OL** | **TST** |
| --- | --- | --- | --- |
| Genome Length(bp) | 2,615,516,299 | 2,615,516,299 | 2,615,516,299 |
| Clean Reads | 144,299,266 | 145,230,879 | 150,397,610 |
| Clean Bases (bp) | 17,952,623,318 | 18,079,425,757 | 18,719,795,555 |
| Mapped Reads | 143,424,496 | 144,399,252 | 149,402,915 |
| Mapping Rate (%) | 99.39387479 | 99.42365348 | 99.33200281 |
| Duplication Rate (%) | 13.55802326 | 14.62172375 | 14.76242823 |
| Uniq Rate (%) | 81.08694259 | 80.32118941 | 80.16880522 |
| Mean Depth | 5.755048135 | 5.846472204 | 5.948248921 |
| Coverage Rate (%) | 94.80449394 | 94.76334627 | 94.92145099 |
| Mismatch Rate (%) | 0 | 0 | 0 |

**Supplementary Table S2.** Numbers and distribution of SNPs in the resequenced sheep breeds .

| Ovis | Number | Percent(%) |
| --- | --- | --- |
| Total | 30356562 | 100 |
| UTR5 | 138076 | 0.45 |
| UTR3 | 177615 | 0.59 |
| UTR5;UTR3 | 106 | 0 |
| exonic | 214098 | 0.71 |
| splicing | 998 | 0 |
| exonic;splicing | 29 | 0 |
| upstream | 168073 | 0.55 |
| downstream | 168365 | 0.55 |
| upstream;downstream | 4120 | 0.01 |
| intronic | 10,599,550 | 34.92 |
| intergenic | 18,839,714 | 62.06 |
| ncRNA_UTR3 | 0 | 0 |
| ncRNA_UTR5 | 0 | 0 |
| ncRNA_exonic | 5,399 | 0.02 |
| ncRNA_splicing | 6 | 0 |
| ncRNA_intronic | 40,412 | 0.13 |
| other | 1 | 0 |

**Supplementary Table S3.** The numbers and percent of nonsynonymous SNV and synonymous SNV in the exons

| Ovis | Number | Percent(%) |
| --- | --- | --- |
| Total | 214098 | 100 |
| nonsynonymous SNV | 62154 | 29.03 |
| synonymous SNV | 81504 | 38.07 |
| stopgain | 794 | 0.37 |
| stoploss | 117 | 0.05 |
| unknown | 69529 | 32.48 |

**Supplementary Table S4.** The numbers and distribution of InDel in the exons.

| Ovis | Number | Percent(%) |
| --- | --- | --- |
| Total | 44557 | 100 |
| frameshift deletion | 4,716 | 10.58 |
| frameshift insertion | 6,968 | 15.64 |
| nonframeshift deletion | 1,182 | 2.65 |
| nonframeshift insertion | 1,736 | 3.9 |
| stopgain | 477 | 1.07 |
| stoploss | 17 | 0.04 |
| unknown | 29,461 | 66.12 |

**Supplementary Table S5.** The proportions of InDels in intergenic, intronic, and exonic of the genome.

| Ovis | Number | Percent(%) |
| --- | --- | --- |
| Total | 5,388,372 | 100 |
| UTR5 | 28,878 | 0.54 |
| UTR3 | 35,060 | 0.65 |
| UTR5;UTR3 | 27 | 0 |
| exonic | 44,557 | 0.83 |
| splicing | 1,320 | 0.02 |
| exonic;splicing | 16 | 0 |
| upstream | 67,763 | 1.26 |
| downstream | 38,672 | 0.72 |
| upstream;downstream | 2,261 | 0.04 |
| intronic | 1,885,711 | 35 |
| intergenic | 3,258,720 | 60.48 |
| ncRNA_UTR3 | 0 | 0 |
| ncRNA_UTR5 | 4 | 0 |
| ncRNA_exonic | 744 | 0.01 |
| ncRNA_splicing | 1 | 0 |
| ncRNA_intronic | 7,516 | 0.14 |
| other | 17,122 | 0.32 |

**Supplementary Table S6.** Estimates of Genetic diversity of three sheep breeds

| Breed | H_O_ | H_E_ | MAF |
| --- | --- | --- | --- |
| TST | 0.25±0.18 | 0.30±0.016 | 0.22±0.14 |
| BZ | 0.23±0.15 | 0.62±0.4 | 0.22±0.14 |
| OL | 0.21±0.17 | 030±0.16 | 0.22±0.15 |
| Over all | 0.24±0.13 | 0.33±0.13 | 0.22±0.13 |

**Supplementary Table S7.** Non-synonymous mutations of overlapping genes in Fst and XP-EHH two methods

| Chromosome | Position | Type | mutation | name | Gene |
| --- | --- | --- | --- | --- | --- |
| chr1 | 20365275 | nonsynonymous | G | A | MAST2 |
| chr1 | 20366938 | nonsynonymous | G | A | MAST2 |
| chr1 | 20369295 | nonsynonymous | G | A | MAST2 |
| chr1 | 20369317 | nonsynonymous | C | T | MAST2 |
| chr1 | 20369487 | nonsynonymous | T | G | MAST2 |
| chr1 | 20369845 | nonsynonymous | A | G | MAST2 |
| chr1 | 63037614 | nonsynonymous | G | A | LOC101116002 |
| chr1 | 63039180 | nonsynonymous | G | A | LOC101116002 |
| chr1 | 63039184 | nonsynonymous | A | G | LOC101116002 |
| chr1 | 63039187 | nonsynonymous | T | C | LOC101116002 |
| chr1 | 63040030 | nonsynonymous | G | T | LOC101116002 |
| chr1 | 63040074 | nonsynonymous | T | G | LOC101116002 |
| chr1 | 63044992 | nonsynonymous | A | G | LOC101116002 |
| chr1 | 63045126 | nonsynonymous | A | G | LOC101116002 |
| chr1 | 63047318 | nonsynonymous | C | A | LOC101116002 |
| chr1 | 63048216 | nonsynonymous | G | A | LOC101116002 |
| chr1 | 63052312 | nonsynonymous | G | A | LOC101116002 |
| chr1 | 63054912 | nonsynonymous | T | A | LOC101116002 |
| chr1 | 63056040 | nonsynonymous | C | T | LOC101116002 |
| chr1 | 63061952 | nonsynonymous | A | C | LOC101116002 |
| chr1 | 63062118 | nonsynonymous | G | A | LOC101116002 |
| chr1 | 183209888 | nonsynonymous | T | C | LRRC58 |
| chr2 | 30944333 | nonsynonymous | T | C | FANCC |
| chr2 | 31033515 | nonsynonymous | A | T | LOC100125610 |
| chr2 | 31033645 | nonsynonymous | T | C | LOC100125610 |
| chr2 | 31033732 | nonsynonymous | A | G | LOC100125610 |
| chr2 | 31033810 | nonsynonymous | G | A | LOC100125610 |
| chr2 | 31033855 | nonsynonymous | C | G | LOC100125610 |
| chr2 | 31033860 | nonsynonymous | G | A | LOC100125610 |
| chr2 | 31033885 | nonsynonymous | T | G | LOC100125610 |
| chr2 | 31033897 | nonsynonymous | G | A | LOC100125610 |
| chr2 | 31033902 | nonsynonymous | A | G | LOC100125610 |
| chr2 | 31034040 | nonsynonymous | G | A | LOC100125610 |
| chr2 | 31034061 | nonsynonymous | C | T | LOC100125610 |
| chr2 | 31034107 | nonsynonymous | T | A | LOC100125610 |
| chr2 | 31034161 | nonsynonymous | A | G | LOC100125610 |
| chr2 | 116597221 | nonsynonymous | C | T | WDR33 |
| chr2 | 116635772 | nonsynonymous | C | T | WDR33 |
| chr2 | 116635787 | nonsynonymous | C | G | WDR33 |
| chr3 | 212931753 | nonsynonymous | G | A | MICAL3 |
| chr3 | 212932024 | nonsynonymous | C | T | MICAL3 |
| chr3 | 212932188 | nonsynonymous | A | G | MICAL3 |
| chr3 | 212932212 | nonsynonymous | A | C | MICAL3 |
| chr3 | 212933115 | nonsynonymous | G | A | MICAL3 |
| chr3 | 212933885 | nonsynonymous | C | T | MICAL3 |
| chr3 | 212937743 | nonsynonymous | G | A | MICAL3 |
| chr3 | 212956022 | nonsynonymous | G | A | MICAL3 |
| chr3 | 212965832 | nonsynonymous | C | T | MICAL3 |
| chr3 | 213103906 | nonsynonymous | C | T | PEX26 |
| chr3 | 213106108 | nonsynonymous | G | A | PEX26 |
| chr3 | 213106154 | nonsynonymous | A | G | PEX26 |
| chr3 | 213111555 | nonsynonymous | C | T | PEX26 |
| chr3 | 213123696 | nonsynonymous | T | G | TUBA8 |
| chr3 | 213503207 | nonsynonymous | C | T | SOX10 |
| chr3 | 213503466 | nonsynonymous | C | T | SOX10 |
| chr3 | 213503496 | nonsynonymous | C | T | SOX10 |
| chr3 | 213503574 | nonsynonymous | G | A | SOX10 |
| chr3 | 213503576 | nonsynonymous | C | T | SOX10 |
| chr3 | 213503607 | nonsynonymous | G | C | SOX10 |
| chr3 | 213506394 | nonsynonymous | G | C | SOX10 |
| chr3 | 213586381 | nonsynonymous | G | A | PICK1 |
| chr3 | 213594142 | nonsynonymous | G | A | PICK1 |
| chr3 | 214161779 | nonsynonymous | G | A | NPTXR |
| chr3 | 214201854 | nonsynonymous | G | C | CBX6 |
| chr3 | 214201868 | nonsynonymous | C | G | CBX6 |
| chr3 | 214201881 | nonsynonymous | C | G | CBX6 |
| chr3 | 214201889 | nonsynonymous | T | G | CBX6 |
| chr4 | 105908663 | nonsynonymous | A | C | LOC101114011 |
| chr4 | 105926382 | nonsynonymous | C | T | LOC101122595 |
| chr4 | 105980722 | nonsynonymous | G | A | LOC105612479 |
| chr4 | 105980936 | nonsynonymous | T | C | LOC105612479 |
| chr4 | 106060540 | nonsynonymous | A | G | GSTK1 |
| chr4 | 106060779 | nonsynonymous | G | A | GSTK1 |
| chr4 | 106084414 | nonsynonymous | G | A | TMEM139 |
| chr4 | 106084517 | nonsynonymous | C | T | TMEM139 |
| chr4 | 106098841 | nonsynonymous | C | A | CASP2 |
| chr4 | 106098846 | nonsynonymous | C | G | CASP2 |
| chr4 | 106098903 | nonsynonymous | A | G | CASP2 |
| chr4 | 106099306 | nonsynonymous | C | A | CASP2 |
| chr5 | 16055092 | nonsynonymous | T | G | FUT5 |
| chr5 | 16066136 | nonsynonymous | G | A | LOC106990135 |
| chr5 | 16066550 | nonsynonymous | G | A | LOC106990135 |
| chr5 | 16066776 | nonsynonymous | C | T | LOC106990135 |
| chr5 | 16099830 | nonsynonymous | C | T | CATSPERD |
| chr5 | 16119610 | nonsynonymous | C | T | CATSPERD |
| chr5 | 16134852 | nonsynonymous | A | G | CATSPERD |
| chr7 | 89369706 | nonsynonymous | G | A | TSHR |
| chr7 | 89370278 | nonsynonymous | C | G | TSHR |
| chr7 | 99897749 | nonsynonymous | T | C | TRIM69 |
| chr7 | 99912288 | nonsynonymous | A | C | TRIM69 |
| chr7 | 99912522 | nonsynonymous | G | C | TRIM69 |
| chr7 | 99916316 | nonsynonymous | G | C | TRIM69 |
| chr7 | 99917260 | nonsynonymous | G | C | TRIM69 |
| chr7 | 99927854 | nonsynonymous | T | C | TRIM69 |
| chr9 | 30860505 | nonsynonymous | G | A | LOC101110341 |
| chr9 | 30860667 | nonsynonymous | T | A | LOC101110341 |
| chr9 | 30860668 | nonsynonymous | A | G | LOC101110341 |
| chr15 | 35426798 | nonsynonymous | T | A | LOC101122667 |
| chr16 | 33696101 | nonsynonymous | A | G | PTGER4 |
| chr17 | 29491841 | nonsynonymous | A | G | MFSD8 |
| chr17 | 29518639 | nonsynonymous | A | C | PLK4 |
| chr17 | 29519431 | nonsynonymous | G | T | PLK4 |
| chr17 | 29519506 | nonsynonymous | G | A | PLK4 |
| chr17 | 29519841 | nonsynonymous | C | T | PLK4 |
| chr17 | 29520601 | nonsynonymous | T | C | PLK4 |
| chr17 | 29522329 | nonsynonymous | T | C | PLK4 |
| chr17 | 29527374 | nonsynonymous | C | T | PLK4 |
| chr17 | 29601462 | nonsynonymous | T | G | HSPA4L |
| chr17 | 29601505 | nonsynonymous | C | A | HSPA4L |
| chr17 | 29601623 | nonsynonymous | C | A | HSPA4L |
| chr17 | 29602063 | nonsynonymous | G | T | HSPA4L |
| chr17 | 29602108 | nonsynonymous | C | T | HSPA4L |
| chr18 | 19312379 | nonsynonymous | A | G | LOC101121887 |
| chr18 | 19312437 | nonsynonymous | A | T | LOC101121887 |
| chr18 | 19312524 | nonsynonymous | A | G | LOC101121887 |
| chr18 | 19312544 | nonsynonymous | A | G | LOC101121887 |
| chr18 | 19312745 | nonsynonymous | A | T | LOC101121887 |
| chr18 | 19312802 | nonsynonymous | C | A | LOC101121887 |
| chr18 | 19312803 | nonsynonymous | T | C | LOC101121887 |
| chr18 | 19312814 | nonsynonymous | A | G | LOC101121887 |
| chr18 | 19312920 | nonsynonymous | A | G | LOC101121887 |
| chr18 | 19312947 | nonsynonymous | A | G | LOC101121887 |
| chr18 | 19312973 | nonsynonymous | G | A | LOC101121887 |
| chr18 | 19313015 | nonsynonymous | G | A | LOC101121887 |
| chr18 | 19313021 | nonsynonymous | G | A | LOC101121887 |
| chr18 | 19313030 | nonsynonymous | A | G | LOC101121887 |
| chr18 | 19313045 | nonsynonymous | A | G | LOC101121887 |
| chr18 | 19313075 | nonsynonymous | T | C | LOC101121887 |
| chr18 | 19313076 | nonsynonymous | G | T | LOC101121887 |
| chr18 | 19313090 | nonsynonymous | G | A | LOC101121887 |
| chr18 | 19364739 | nonsynonymous | G | A | LOC101122633 |
| chr18 | 19364747 | nonsynonymous | T | G | LOC101122633 |
| chr18 | 19364841 | nonsynonymous | G | A | LOC101122633 |
| chr18 | 19364844 | nonsynonymous | C | T | LOC101122633 |
| chr18 | 19364946 | nonsynonymous | C | T | LOC101122633 |
| chr18 | 19365147 | nonsynonymous | G | T | LOC101122633 |
| chr18 | 19365159 | nonsynonymous | C | T | LOC101122633 |
| chr18 | 19365170 | nonsynonymous | G | A | LOC101122633 |
| chr18 | 41785164 | nonsynonymous | G | C | AKAP6 |
| chr18 | 41785553 | nonsynonymous | A | G | AKAP6 |
| chr18 | 41786669 | nonsynonymous | T | C | AKAP6 |
| chr18 | 41786708 | nonsynonymous | T | A | AKAP6 |
| chr18 | 42017714 | nonsynonymous | C | T | AKAP6 |
| chr18 | 42062054 | nonsynonymous | C | A | AKAP6 |
| chr18 | 42062622 | nonsynonymous | A | C | AKAP6 |
| chr18 | 42062622 | nonsynonymous | A | G | AKAP6 |
| chr18 | 42063368 | nonsynonymous | C | A | AKAP6 |
| chr18 | 42063505 | nonsynonymous | C | A | AKAP6 |
| chr18 | 42063956 | nonsynonymous | G | A | AKAP6 |
| chr18 | 42064061 | nonsynonymous | G | A | AKAP6 |
| chr18 | 42064353 | nonsynonymous | C | G | AKAP6 |
| chr18 | 42064523 | nonsynonymous | A | G | AKAP6 |
| chr18 | 42064578 | nonsynonymous | G | A | AKAP6 |
| chr18 | 42064633 | nonsynonymous | C | G | AKAP6 |
| chr18 | 42064710 | nonsynonymous | C | T | AKAP6 |
| chr20 | 49518401 | nonsynonymous | G | T | SERPINB1 |
| chr20 | 49565515 | nonsynonymous | A | T | MYLK4 |
| chr20 | 49565545 | nonsynonymous | G | A | MYLK4 |
| chr20 | 49626036 | nonsynonymous | C | T | MYLK4 |
| chr22 | 6728925 | nonsynonymous | C | T | PRKG1 |
| chr22 | 8088835 | nonsynonymous | G | T | PRKG1 |
| chr22 | 8088838 | nonsynonymous | G | T | PRKG1 |
| chr22 | 8088846 | nonsynonymous | G | C | PRKG1 |
| chr22 | 8088849 | nonsynonymous | G | A | PRKG1 |
| chr22 | 8088850 | nonsynonymous | G | T | PRKG1 |
| chr22 | 8088852 | nonsynonymous | G | T | PRKG1 |
| chr22 | 8088856 | nonsynonymous | G | T | PRKG1 |
| chr22 | 8088859 | nonsynonymous | C | A | PRKG1 |
| chr22 | 8088867 | nonsynonymous | G | C | PRKG1 |
| chr22 | 8088877 | nonsynonymous | G | T | PRKG1 |
| chr22 | 8088880 | nonsynonymous | G | T | PRKG1 |
| chr22 | 8089077 | nonsynonymous | G | T | PRKG1 |
| chr22 | 8089081 | nonsynonymous | A | T | PRKG1 |

**Supplementary Table S8.** GO nrichment Analysis for candidate genes

| GO term | GO category | Gene number | p-value | Gene name |
| --- | --- | --- | --- | --- |
| GO:1901698 | response to nitrogen compound | 15 | 0.000202626 | DRD3;MTOR;SFRP1;SLC2A4;KAT2B;PDPK1;TNFAIP3;PDXP;LIN28B;BAD;NDEL1;  PHIP;PDE1B;MAVS;DGKQ |
| GO:0070555 | response to interleukin-1 | 5 | 0.001024998 | IRF1;MAST2;TIGIT |
| GO:0001501 | skeletal system development | 13 | 0.001646063 | HOXC11;ASH1L;GNAQ;HOXC10;HOXC4;SFRP1;ALX4;HHIP;HOXC8;HIF1A;HOXC9;  HOXC5;HOXC6 |
| GO:0009790 | embryo development | 22 | 0.001835826 | SFRP1;MYH10;KDM2B;HOXC11;NDEL1;PRPF19;HOXC10;ALX4;EXT2;DVL2;TSHR;  ARFRP1;CECR2;GLMN;HOXC9;HOXC4;NKX3-1;GNAQ;NKX2-6;HOXC5;HOXC6 |
| GO:0048598 | embryonic morphogenesis | 15 | 0.003238612 | HIF1A;SFRP1;KDM2B;HOXC11;HOXC10;ALX4;EXT2;DVL2;TSHR;ARFRP1;CECR2;  GLMN;HOXC9;HOXC4;GNAQ |
| GO:0002682 | regulation of immune system process | 21 | 0.005460062 | SFRP1;BAD;TNFAIP3;NCKAP1L;GLMN;CCL25;TKFC;RIPK1;CCDC88B;CD6;LGALS1;  MAVS;HIF1A;IRF1;PLCB1;ZBTB46;PDPK1;TYRO3;TIGIT;MITF;MTOR |
| GO:0055013 | cardiac muscle cell development | 3 | 0.010568641 | NKX2-6;MTOR;MYH10 |
| GO:0090102 | cochlea development | 3 | 0.011608343 | DVL2;TSHR;CECR2 |
| GO:0050951 | sensory perception of temperature stimulus | 2 | 0.012506822 | KCNK4;TRPA1 |
| GO:0043586 | tongue development | 2 | 0.012506822 | NKX2-6;HOXC13 |
| GO:0048565 | digestive tract development | 5 | 0.014066493 | PTK6;HIF1A;SFRP1;ALX4;NKX2-6 |
| GO:0007632 | visual behavior | 3 | 0.02518598 | DRD3;HIF1A;PDE1B |
| GO:0045088 | regulation of innate immune response | 6 | 0.032991443 | TNFAIP3;TKFC;MAVS;IRF1;PDPK1;TYRO3 |
| GO:0097411 | hypoxia-inducible factor-1alpha signaling pathway | 1 | 0.046804881 | HIF1A |
| GO:0021591 | ventricular system development | 2 | 0.046894076 | KDM2B;MYH10 |
| GO:0032870 | cellular response to hormone stimulus | 8 | 0.048270393 | ESRRA;SFRP1;SLC2A4;KAT2B;PDPK1;NKX3-1;NDEL1;PHIP |
| GO:0010573 | vascular endothelial growth factor production | 1 | 0.048635601 | HIF1A;FLT |
| GO:0001942 | hair follicle developmen | 4 | 0.008769046 | FGF7;INTU;ALX4;DKK1 |
| GO:0030318 | melanocyte differentiation | 2 | 0.019943743 | KIT;MITF |
| GO:0060173 | limb development | 5 | 0.044184194 | DKK1;ALX4;INTU;RARB;GNAQ |

**Supplementary Table S9.** KEGG Pathway Enrichment Analysis for candidate genes

| map | p-value | name | Gene |
| --- | --- | --- | --- |
| map04740 | 0.000411699 | Olfactory transduction | LOC101102421; CAMK2A; LOC106990711 |
| map00300 | 0.001227126 | Lysine biosynthesis | AADAT; ACO1 |
| map04910 | 0.002830605 | Insulin signaling pathway | BAD; PRKAR2A; PRKAA1; CBL; SLC2A4; PPP1CB; CBLB |
| map05200 | 0.007542361 | Pathways in cancer | CREBBP; CHUK; BAD; VEGFB; VEGFA; CBL; LAMC2; LAMC1; DVL2; PTCH1; CBLB |
| map05160 | 0.009770277 | Hepatitis C | CHUK; IRF7; BAD; RIPK1; CLDN7; IRF1 |
| map05020 | 0.009928067 | Prion diseases | STIP1; LAMC1; LOC101115136 |
| map04310 | 0.013431137 | Wnt signaling pathway | SFRP1; CREBBP; FOSL1; PLCB3; DVL2; CAMK2A |
| map04720 | 0.013648603 | Long-term potentiation | CREBBP; PLCB3; CAMK2A; PPP1CB |
| map05220 | 0.016744112 | Chronic myeloid leukemia | CHUK; BAD; CBL; CBLB |
| map04920 | 0.017581141 | Adipocytokine signaling pathway | CHUK; PRKAA1; TNFRSF1B; SLC2A4 |
| map04622 | 0.020247394 | RIG-I-like receptor signaling pathway | CHUK; IRF7; RIPK1; NLRX1 |
| map04668 | 0.021504781 | TNF signaling pathway | CHUK; RPS6KA4; RIPK1; TNFRSF1B; TNFAIP3 |
| map05410 | 0.023150499 | Hypertrophic cardiomyopathy (HCM) | PRKAA1; CACNG1; CACNG4; CACNG5 |
| map04510 | 0.02460823 | Focal adhesion | BAD; VEGFB; VEGFA; ARHGAP5; LAMC2; LAMC1; PPP1CB |
| map04012 | 0.025219769 | ErbB signaling pathway | BAD; CBL; CAMK2A; CBLB |
| map01210 | 0.027416628 | 2-Oxocarboxylic acid metabolism | AADAT; ACO1 |
| map05205 | 0.032511757 | Proteoglycans in cancer | VEGFA; CBL; CAMK2A; CD63; PPP1CB; PTCH1; CBLB |
| map04064 | 0.038561273 | NF-kappa B signaling pathway | CHUK; RIPK1; LYN; TNFAIP3 |
| map04621 | 0.039262475 | NOD-like receptor signaling pathway | CHUK; CARD6; TNFAIP3 |
| map04210 | 0.039940833 | Apoptosis | CHUK; BAD; RIPK1; PRKAR2A |
| map04730 | 0.045193332 | Long-term depression | PLCB3; LYN; GRID2 |
| map04623 | 0.045193332 | Cytosolic DNA-sensing pathway | CHUK; IRF7; RIPK1 |
| map05014 | 0.049380552 | Amyotrophic lateral sclerosis (ALS) | BAD; TNFRSF1B; LOC101115136 |
| map04725 | 0.012445625 | Cholinergic synapse | CAMK2G; KCNQ3; KCNQ5; CREB3 |
| map00511 | 0.014289931 | Other glycan degradation | MAN2C1; GBA2 |
| map05031 | 0.017561284 | Amphetamine addiction | CAMK2G; LOC101117953; CREB3 |
| map00270 | 0.034344739 | Cysteine and methionine metabolism | LOC101122577; AHCYL1 |
| map04740 | 0.05619766 | Olfactory transduction | CAMK2G; LOC101114017; LOC101107959 |

**Supplementary Table S10:** the list of 45 genes under selection across the three breeds

| Gene name | | | | |
| --- | --- | --- | --- | --- |
| RAD54L | SNUPN | CLIP1 | RNH1 | RCOR1 |
| POMGNT1 | PTPN9 | ATG2A | LOC101102851 | KNTC1 |
| TSPAN1 | CDC42BPG | LRRC41 | FAAH | SLC25A35 |
| PIK3R3 | LMNTD2 | C18H15orf39 | LOC101103602 | PANK2 |
| UQCRH | CUX1 | ODF4 | FAM20C | RNF24 |
| SPAG16 | PFAS | PTDSS2 | RSRC2 | LRRC43 |
| RFX3 | LURAP1 | KRBA2 | EHD1 | MLXIP |
| LEF1 | NSUN4 | RPL26 | LRRC56 | LOC101114579 |
| TRMT11 | RANGRF | HLTF | HRAS | ZCCHC8 |

**Supplementary Table S11:** Description of three sheep breeds in the study

| Breeds | Qinghai Tibetan Sheep (BZ) | Poll Dorst sheep (TST) | Oula sheep (OL) |
| --- | --- | --- | --- |
| Coat Colour | White | White | Mainly white with grey pigmentation |
| Body size | Small | Large | Medium |
| wool lengths | Long wool(26.37±1.18cm) | Medium wool(7.5-10.5cm) | Short wool |
| Uses | Mainly Wool | Meat | Meat |
| Community | Qinghai/Xining | Qinghai/Xining | Qinghai/Huangnan |
| Breeds type | Native breed | Imported breed | Native breed |

**Supplementary Figure S1.** Comparison of mean corpuscular hemoglobin concentration (MCHC) and mean corpuscular hemoglobin measurement (MCH) from three sheep breeds


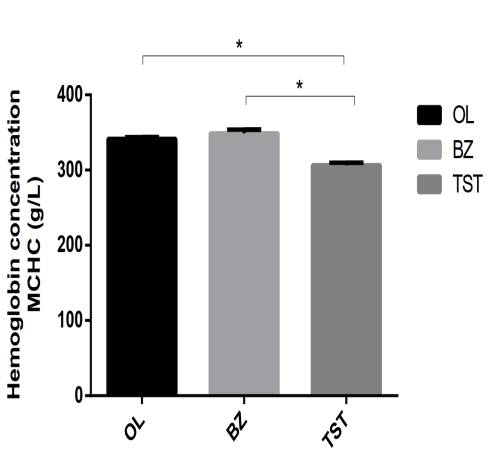

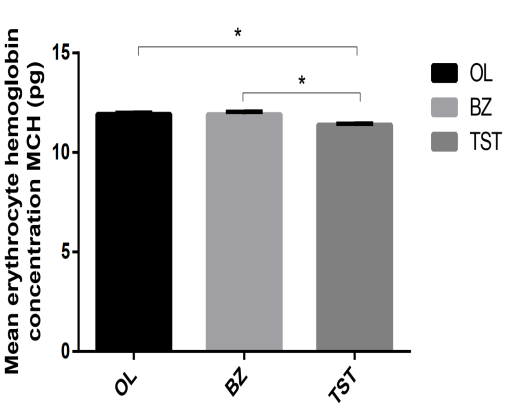


**b**

**a**

**Supplementary Figure S2.** Analysis of the signatures of positive selection in the genome of samples. Manhattan plot of Genomic landscape of the XP-EHH P-values


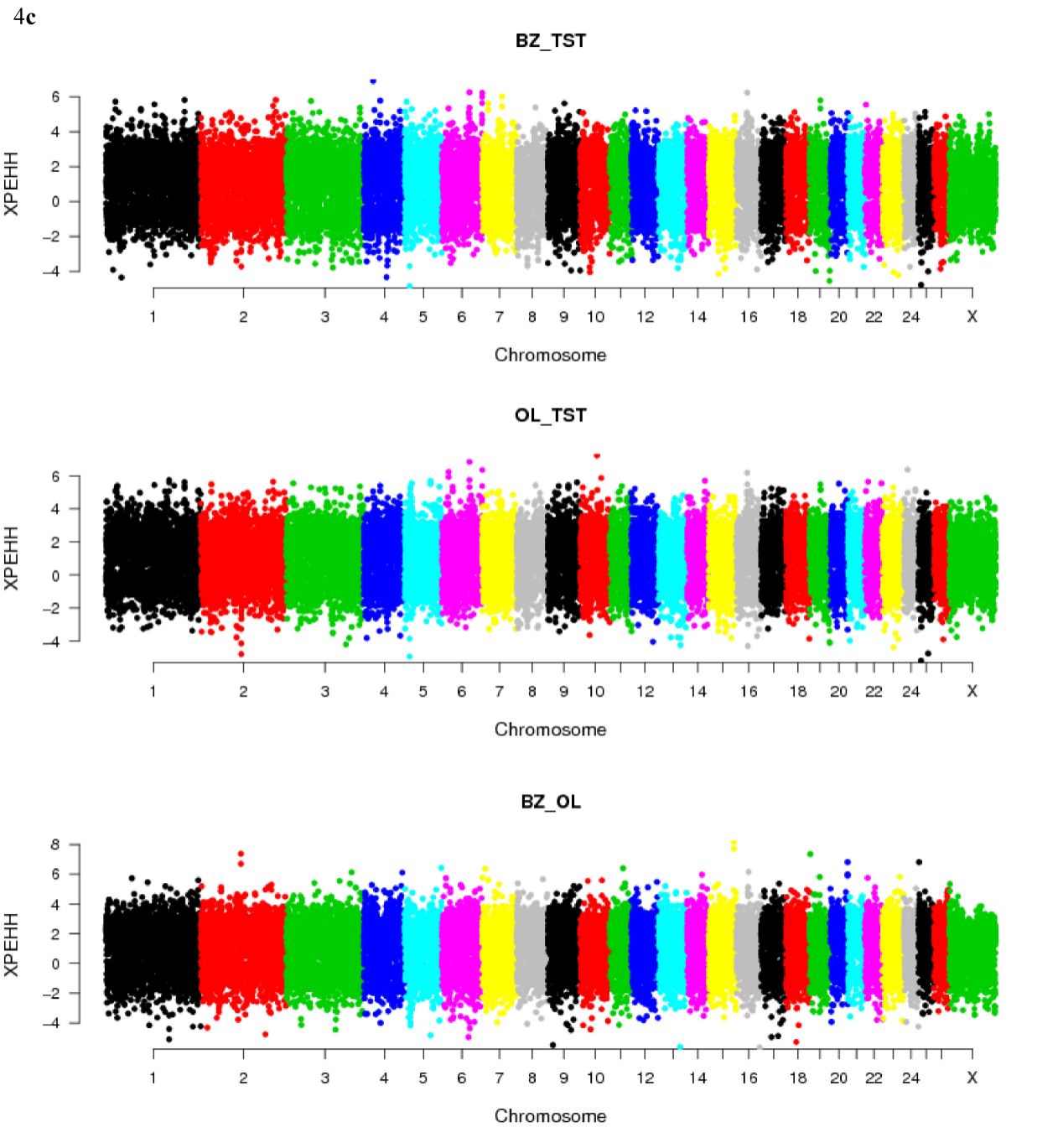

Supplement: Supplementary file 1 — Supplementary Information [file 41598_2021_81932_MOESM1_ESM.docx]
